# Multi Omics Integration in Colorectal Cancer: From Molecular Insights to Precision Oncology

**DOI:** 10.3390/cancers18101504

**Published:** 2026-05-07

**Authors:** Zuoliang Liu, Mia Yang Ang, Chin Siang Kue

**Affiliations:** 1 Department of Gastrointestinal Surgery, Affiliated Hospital of North Sichuan Medical College, Maoyuan South Road, Shunqing District, Nanchong 637000, China; liuzuoliang@nsmc.edu.cn; 2 School of Graduate Studies, Post Graduate Centre, Management and Science University, Seksyen 13, Shah Alam 40100, Selangor, Malaysia; 3 Institute of Hepatobiliary Pancreatic Intestinal Diseases, North Sichuan Medical College, Maoyuan South Road, Shunqing District, Nanchong 637000, China; 4 National Clinical Key Specialty (General Surgery), Sub-Center of National Clinical Research Center for Digestive Diseases, Sichuan Clinical Research Center for Digestive Diseases, Nanchong 637000, China; 5 Department of Diagnostic and Allied Health Science, Faculty of Health and Life Sciences, Management and Science University (MSU), University Drive, Off Persiaran Olahraga, Section 13, Shah Alam 40100, Selangor, Malaysia

**Keywords:** colorectal cancer, multi-omics integration, proteogenomic, gut microbiota, consensus molecular subtypes, liquid biopsy, biomarker discovery, spatial transcriptomics, artificial intelligence

## Abstract

Colorectal cancer (CRC) is a biologically heterogeneous disease in which single-omics analyses incompletely capture the cross-layer mechanisms underlying tumor progression, immune evasion, and therapeutic resistance. This review critically examines how the integration of genomics, transcriptomics, proteomics, metabolomics, and microbiome profiling is redefining CRC biology and precision oncology. Landmark integrative efforts, including TCGA analyses of 276 colorectal cancer samples, CPTAC proteogenomic profiling of 95 tumors, and recent whole-genome sequencing studies of 2023 CRC cases, have refined molecular subtyping, expanded the driver landscape, and revealed clinically relevant discordance between mRNA abundance and protein activity. Integrative studies further show that oncogenic signaling may be driven by post-transcriptional and post-translational regulation, while spatially resolved profiling and microbiome–metabolite analyses are uncovering previously obscured tumor–microenvironment interactions. We also discuss how artificial intelligence-based approaches, including factor analysis, deep learning, graph-based models, and explainable AI, are improving subtype classification, biomarker discovery, and treatment-response prediction, with particular relevance to microsatellite instability-high and early-onset CRC. Finally, we critically evaluate the principal barriers to clinical translation, including batch effects, cross-platform variability, limited external validation, regulatory constraints, and cost, and outline priorities for building reproducible, clinically deployable multi-omics pipelines for CRC management.

## Simple Summary

Colorectal cancer (CRC) is not a single disease but a complex condition shaped by genetic changes, altered cell signaling, metabolism, and interactions with intestinal microbes. Traditional approaches that examine only one biological layer at a time often miss how these factors work together. Multi-omics research combines information from genes, RNA, proteins, metabolites, and the microbiome to provide a more complete picture of tumor behavior. This review explains how such integrated approaches are improving disease classification, biomarker discovery, and treatment selection, including for immunotherapy and early-onset colorectal cancer. It also highlights the growing role of artificial intelligence in analyzing these complex datasets. At the same time, important barriers remain, such as limited standardization, high cost, and difficulties in translating research findings into routine clinical care. Understanding these advances and challenges may help guide more precise and practical colorectal cancer management in the future.

## 1. Introduction

Colorectal cancer (CRC) remains one of the most frequently diagnosed malignancies and a major cause of cancer-related death worldwide. In 2022, there were an estimated 1,926,425 new cases and 904,019 deaths, underscoring the continuing need for better strategies in detection, risk stratification, and treatment [1]. Colonoscopy, fecal immunochemical testing, and computed tomography colonography remain central to CRC screening [2]. However, the main limitations of precision oncology in CRC extend beyond screening alone. In routine practice, treatment decisions still rely on a relatively narrow set of clinicopathological parameters and selected molecular markers. However, patients with apparently similar profiles often show markedly different responses, patterns of resistance, and clinical outcomes. Current approaches therefore do not fully account for the biological diversity of CRC or its evolution over time.

This problem is especially relevant in the context of early-onset colorectal cancer (EOCRC), usually defined as CRC diagnosed before the age of 50 [3]. Although hereditary syndromes remain important in a subset of patients, they explain only a minority of EOCRC cases [4]. This suggests that broader environmental and lifestyle-related influences are contributing to the recent rise in incidence. Factors such as Westernized dietary patterns, obesity, physical inactivity, antibiotic exposure, and other early-life perturbations have all been proposed as possible contributors [5]. Increasing attention has also been directed toward the gut microbiome. Younger patients may harbor altered microbial communities and microbe-derived metabolites that influence mucosal inflammation, epithelial barrier integrity, and carcinogenic signaling. Emerging metabolomic evidence further suggests that EOCRC may involve changes in bile acid metabolism, short-chain fatty acid balance, and amino acid utilization [6]. Taken together, these observations suggest that EOCRC is not simply CRC occurring at a younger age. Instead, it may reflect a distinct biological setting in which environmental, microbial, and metabolic factors play a greater role [7]. CRC itself is shaped by interactions across multiple biological layers, including genomic alterations, transcriptional programs, proteomic signaling, metabolic rewiring, and host–microbiome dynamics [8]. Single-omics studies have provided an important foundation. They identify recurrent driver mutations such as *APC*, *KRAS*, and *TP53* and supporting classification systems such as the Consensus Molecular Subtypes (CMS). Even so, these approaches capture only part of the disease process and often do not explain how different molecular layers converge to influence progression, immune escape, or treatment resistance. Landmark integrative studies helped shift this view. Work from The Cancer Genome Atlas (TCGA) established a foundational molecular framework for CRC by integrating genomic and transcriptomic features [9]. Studies from the Clinical Proteomic Tumor Analysis Consortium (CPTAC) have shown that clinically relevant protein activity and phosphorylation-based signaling states are not always directly reflected by mRNA abundance. These findings made it increasingly clear that multi-omics integration is not merely an expansion of profiling depth. It is a more appropriate framework for studying a disease as heterogeneous as CRC [10]. Several major questions still remain unresolved. How tumor-intrinsic alterations interact with the immune and stromal microenvironment cannot be fully understood without integrating genomic, transcriptomic, proteomic, and spatial information. Likewise, the variability observed within established CRC subtypes suggests that transcriptional classifications alone are insufficient. Additional proteomic, metabolic, and microbiome layers may help explain clinically divergent behavior. Therapeutic resistance also tends to emerge through coordinated changes across multiple biological levels rather than through a single molecular event. In addition, robust biomarkers for early detection, prognosis, and longitudinal monitoring will likely require composite, multi-layered signatures rather than isolated markers. Viewed in this way, multi-omics integration is not simply a broader profiling strategy. Rather, it is a necessary framework for addressing key unresolved questions in CRC biology and precision oncology.

**Figure 1:**
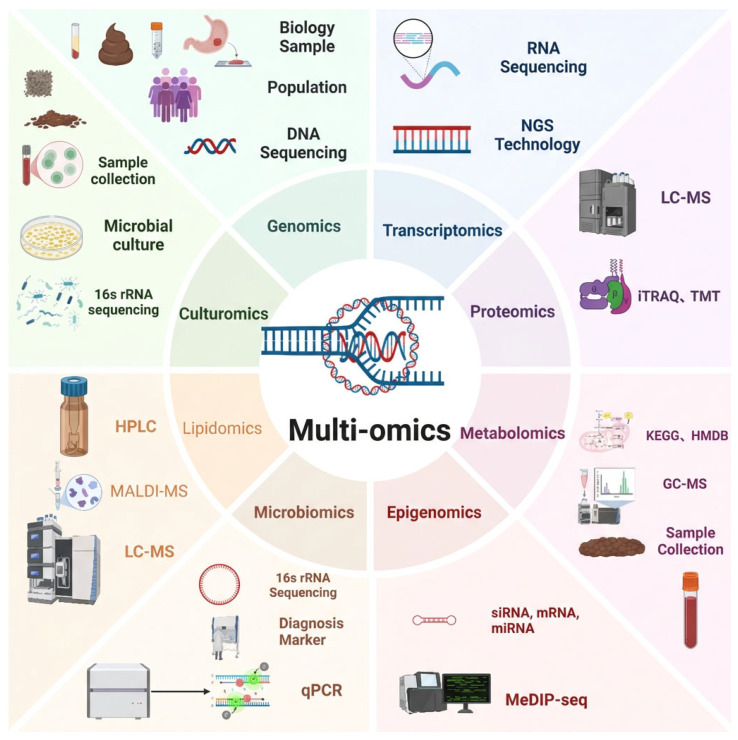
Integrative multi-omics framework for CRC research. Multi-omics profiling (genomics, transcriptomics, proteomics, metabolomics, metagenomics) enables unified molecular models informing biomarker discovery, therapeutic target identification, and precision medicine applications.

In this review, we examine how integrated genomic, transcriptomic, proteomic, metabolomic, and microbiome analyses are reshaping current understanding of CRC biology, molecular classification, and clinical stratification. We also highlight translational challenges and the growing relevance of EOCRC. Despite substantial progress, formidable obstacles remain. Data standardization, cohort heterogeneity, computational integration frameworks, and rigorous clinical validation continue to hinder routine clinical implementation. In addition, large-scale, demographically diverse, and longitudinally characterized patient cohorts, including adequate EOCRC representation, are urgently needed for robust biomarker validation and mechanistic investigation (Figure 1).

## 2. Genomics: The Landscape of Driver Alterations and Molecular Subtypes

### 2.1. Key Genomic Drivers and Pathways

**Figure 2:**
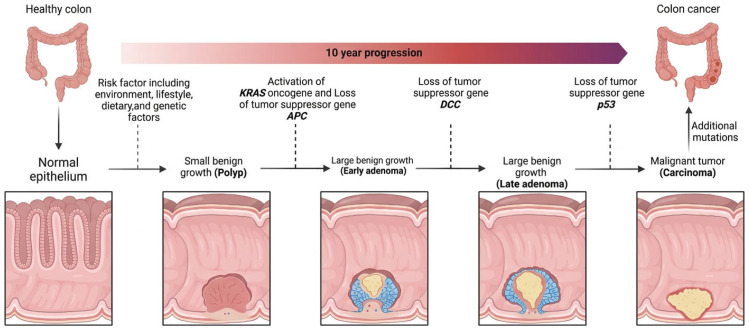
Classical adenoma–carcinoma sequence in colorectal cancer. Sequential accumulation of canonical driver alterations, including *APC*, *KRAS*, and *TP53*, and the associated histopathological progression from normal epithelium to invasive carcinoma. Although this model remains a useful framework, alternative routes of CRC development, including serrated and mismatch repair-deficient pathways, are also recognized.

Driver mutations confer selective growth advantages propelling tumor initiation and progression. They are distinct from passenger mutations that accumulate passively. In CRC, the mutational landscape is dominated by alterations in the WNT signaling pathway, particularly APC (~80%) [11]. Frequent mutations are also found in RAS–MAPK pathway (KRAS mutations in ~40–45%, BRAF V600E in ~10–15%) [12,13], PI3K–AKT pathway (PIK3CA mutations in ~15–20%) [14], and TP53 tumor suppressor pathway (mutations in ~50–60% of cases) [15] (Table 1). These mutations drive the sequential adenoma-to-carcinoma progression (Figure 2).

Although the adenoma–carcinoma sequence remains a useful conceptual model for CRC development, it does not capture the full diversity of tumorigenic routes. A substantial subset of CRCs arises through alternative pathways, most notably the serrated neoplasia pathway. This pathway is commonly associated with sessile serrated lesions, BRAF mutation, and CpG island methylator phenotype (CIMP). In some cases, it is also linked to subsequent mismatch repair deficiency and microsatellite instability [21]. In parallel, hypermutated CRCs may develop through mismatch repair-deficient mechanisms that do not conform to the classical stepwise *APC–KRAS–TP53* progression [22]. More recent genomic studies also suggest that CRC evolution is often non-linear, with branching trajectories shaped by chromosomal instability, epigenetic remodeling, and structural variation [23]. Taken together, these observations indicate that Figure 2 should be viewed as a representative model rather than a universal pathway for all CRCs.

APC mutations disrupt the β-catenin destruction complex. This leads to constitutive WNT activation. Mutation location also affects phenotype. Truncating mutations in the mutation cluster region are associated with intermediate WNT activity and a more favorable prognosis. By contrast, mutations outside this region are linked to higher WNT activity and more aggressive disease [24]. KRAS mutations (codons 12, 13, 61) constitutively activate MAPK and PI3K signaling. However, different KRAS variants are not biologically equivalent. Functional studies have shown that specific substitutions such as G12D and G12V can differ in signaling output. They also differ in therapeutic vulnerability, including differential sensitivity to MEK pathway inhibition [25]. By contrast, G13D shows distinct biochemical behavior. It may also retain altered interactions with regulatory proteins such as NF1. This may partly explain its atypical response to EGFR inhibition [26]. KRAS mutations predict anti-EGFR therapy resistance (cetuximab, panitumumab), mandating testing before treatment [12]. BRAF V600E mutations (~10–15%) confer poor prognosis in microsatellite-stable tumors. They are also associate with right-sided location and CMS1 subtype, and may show modest benefit from encorafenib plus cetuximab combination therapy [27]. TP53 mutations (~50–60%) arise during late adenoma-to-carcinoma transition. Missense mutations in the DNA-binding domain exhibit gain-of-function properties, promoting invasion, metastasis, and chemoresistance. Emerging evidence also links TP53 mutation status to chemotherapy response [28]. PIK3CA mutations (~15–20%) activate PI3K–AKT–mTOR signaling and frequently co-occur with KRAS mutations. Although PIK3CA inhibitors have shown limited CRC efficacy, these mutations may predict aspirin chemoprevention benefit (Table 2) [29].

Beyond these canonical driver events, recent whole-genome sequencing studies have substantially expanded the genomic framework of colorectal cancer [30]. In particular, large-scale analysis of 2023 CRCs from the UK 100,000 Genomes Project provided a high-resolution map of somatic alterations [23] and identified more than 250 putative driver genes. Many of these genes had not previously been implicated in CRC or, in some cases, in cancer more broadly. These data also refined the mutational processes operating in CRC and extended the set of molecular pathways involved in tumor development. Importantly, the study detected recurrent alterations outside the coding genome, highlighting the likely contribution of non-coding regulatory mutations that may influence transcriptional control and tumor behavior. Whole-genome sequencing also captures structural variation more effectively than targeted or exome-based approaches, including large-scale rearrangements, copy-number changes, and other chromosomal alterations that contribute to genomic instability and inter-tumoral heterogeneity. Together, these findings indicate that CRC genomics cannot be fully understood through a coding-centric view alone. More recent integrative genome–transcriptome analyses of over 1000 primary CRCs have reinforced this shift by linking newly identified driver events to functional and prognostic consequences [30]. International whole-genome studies further suggest that mutational processes vary according to geography and age. These observations are especially relevant to current efforts to refine molecular subtypes and explain biological diversity within clinically similar tumors. They are also important for linking genomic architecture with downstream transcriptomic and proteomic states [31].

**Table 2:** Prevalence, roles and references of key genetic alterations in CRC. The table summarizes frequently mutated genes in CRC, their occurrence rates, functional impact on tumor biology, and associated literature.

Gene	Type	Prevalence	Role in CRC	Ref.
APC	Tumor suppressor	∼80% of sporadic cases	Initiating event in tumorigenesis; regulates β-catenin	[32]
KRAS	Oncogene	~40–45% of cases	Early event in adenoma-carcinoma sequence; guides anti-EGFR therapy	[12]
TP53	Tumor suppressor	∼50–60% of cases	Maintains genomic stability; prevalent alteration in CRC	[15]
PIK3CA	Oncogene	∼15–20% of cases	Implicated in resistance to anti-EGFR therapy; involved in PI3K pathway	[14]
BRAF V600E	Oncogene	∼10–15% of cases	Mutations affect MAPK pathway; associated with aggressive disease	[13]

Abbreviations: CRC, colorectal cancer; EGFR, epidermal growth factor receptor; PI3K, phosphatidylinositol 3-kinase; MAPK, mitogen-activated protein kinase; APC, adenomatous polyposis coli; TP53, tumor protein p53.

**Figure 3:**
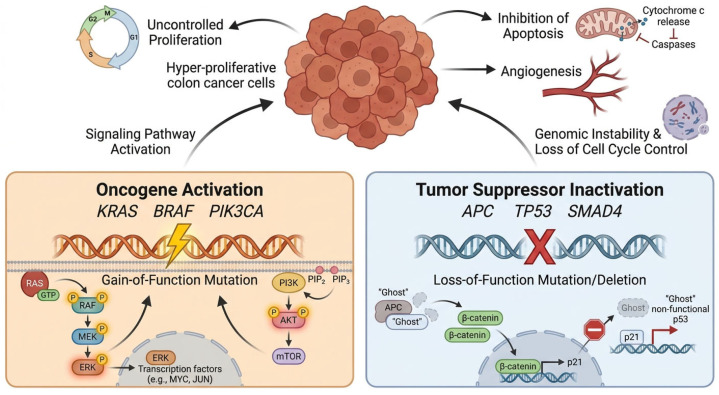
Classification and mechanisms of driver genes in CRC. Key driver genes categorized as oncogenes (orange) and tumor suppressor genes (blue), showing representative examples and their molecular mechanisms in tumor initiation and progression.

Recent whole-genome sequencing of 2023 CRC specimens identified over 250 putative driver genes (Figure 3), including subtype-specific candidates [23]. Integrated multi-omics analyses have resolved temporal driver events and established molecular subtypes defined by Wnt, EGFR, and TGF-β signaling engagement [33]. Epigenetic regulators, including ATRX (~7% of CRCs), also contribute to CRC progression [34]. They may promote metastasis through epithelial-to-mesenchymal transition and chromatin remodeling, revealing potentially actionable therapeutic targets for precision intervention.

### 2.2. Microsatellite Instability (MSI) as a Biomarker Paradigm

Microsatellite instability (MSI) results from defective DNA mismatch repair (MMR). It is typically caused by biallelic inactivation of MLH1, MSH2, MSH6, or PMS2 through germline mutations (Lynch syndrome, ~3% of CRCs) or somatic MLH1 promoter hypermethylation (sporadic cases, ~12%) [35]. MSI-high (MSI-H) tumors exhibit a hypermutator phenotype. They often show frameshift mutations in TGFBR2 and BAX, right-sided location, mucinous histology, and prominent immune infiltration. High neoantigen burden drives robust antitumor immunity, conferring favorable stage-adjusted survival. However, BRAF V600E co-mutations may worsen outcomes.

Beyond its biological significance, MSI has direct clinical utility across several stages of colorectal cancer management. Universal MMR/MSI testing is now recommended at diagnosis in NCCN guideline for colon cancer. This helps identify patients with possible Lynch syndrome and guides treatment planning. In localized disease, dMMR/MSI-H status is particularly relevant in stage II colon cancer. These tumors generally have a more favorable stage-adjusted prognosis and do not derive meaningful benefit from fluoropyrimidine monotherapy [36]. The NCCN guideline also now includes dedicated biomarker-stratified pathways for dMMR/MSI-H rectal cancer, including checkpoint inhibitor-based strategies in selected locally advanced and metastatic settings. In advanced disease, MSI-H/dMMR has become one of the most established predictive biomarkers for immune checkpoint blockade [37]. In the 5-year follow-up of the phase III KEYNOTE-177 trial, 307 patients with previously untreated MSI-H/dMMR metastatic colorectal cancer were randomized to pembrolizumab (n = 153) or chemotherapy (n = 154). Median overall survival was 77.5 versus 36.7 months, 5-year overall survival was 54.8% versus 44.2%. Median progression-free survival was 16.5 versus 8.2 months and median duration of response was 75.4 versus 10.6 months [38]. More recent phase III data from CheckMate 8HW further support first-line nivolumab plus ipilimumab in this setting. Among 303 randomized patients, including 255 with centrally confirmed MSI-H/dMMR tumors, 24-month progression-free survival was 72% with nivolumab plus ipilimumab compared with 14% with chemotherapy. Grade 3 or 4 treatment-related adverse events occurred in 23% versus 48% of patients, respectively [39]. Together, these applications make MSI one of the clearest examples of how molecular classification has already entered routine clinical decision-making in CRC.

### 2.3. Consensus Molecular Subtypes (CMS)

Integration of genomic and transcriptomic data has classified CRC into four biologically distinct subtypes with therapeutic implications [40]. CMS1 (MSI-Immune, ~14%) exhibits hypermutation, immune activation, frequent BRAF mutations, and responsiveness to immunotherapy. CMS2 (Canonical, ~37%) shows epithelial differentiation, WNT/MYC activation, and chromosomal instability. CMS3 (Metabolic, ~13%) displays metabolic dysregulation with frequent KRAS mutations. CMS4 (Mesenchymal, ~23%) demonstrates TGF-β activation, stromal invasion, epithelial–mesenchymal transition, and poor prognosis. Recent multi-omics and spatial analyses indicate that CMS4 is not solely a tumor-intrinsic transcriptional state. Instead, it reflects a stromal-rich microenvironment characterized by activated cancer-associated fibroblasts, TGF-β signaling, and immune exclusion [41]. This framework provides a foundation for understanding CRC heterogeneity. Ongoing multi-omics studies are further refining subtype boundaries and clinical utility (Table 3).

## 3. Transcriptomics: From Bulk Profiling to Spatial Resolution

Transcriptomics profiles RNA transcripts to link genomic alterations with functional outcomes in CRC. By quantifying mRNA and non-coding RNAs, it delineates gene-expression dynamics, regulatory networks, and disease-specific signatures, enabling biomarker discovery for diagnosis, prognosis, and therapy. This section highlights key advances in transcriptomic profiling, the roles of non-coding RNAs, and their translational applications in CRC [42].

### 3.1. Gene Expression Signatures and Biomarkers

Transcriptome analysis, led by RNA-seq, has redefined the gene-expression landscape in CRC. Comparison of tumor and normal tissues reveals differentially expressed genes (DEGs) involved in cancer hallmarks, including cell-cycle dysregulation, immune evasion, and EMT [43,44]. Overexpression of genes like *MMP7*, *S100A4*, and *CEACAM5* is consistently linked to invasion, metastasis, and poor prognosis [45]. Expression signatures further stratify patients into molecular subgroups and complement genomic classifications such as CMS (Table 4).

Interpretation of RNA-seq data in CRC also depends heavily on the computational pipeline used. In most bulk RNA-seq studies, raw reads first undergo quality control and filtering, followed by adapter trimming and alignment to the reference genome or pseudoalignment to the transcriptome. Gene- or transcript-level quantification is then generated and subjected to normalization before differential expression analysis, clustering, and downstream functional interpretation. Depending on the aims of the study, these data may also be combined with pathway enrichment, co-expression network analysis, immune deconvolution, or integrative multi-omics modeling. In single-cell and spatial transcriptomic datasets, further computational steps are usually required, including cell filtering, batch correction, dimensionality reduction, clustering, trajectory inference, and ligand–receptor or cell–cell communication analysis. These analytical choices are not merely technical details, as they can substantially influence subtype assignment, biomarker discovery, and biological interpretation in CRC [58,59].

In CRC, transcriptomic studies have identified gene expression signatures associated with proliferation, epithelial–mesenchymal transition, immune exclusion, stromal activation, and metastatic potential. Such signatures have helped refine molecular stratification beyond mutational status alone and have highlighted why tumors with similar genomic profiles may still behave differently in clinical practice. Transcriptomic biomarkers are therefore increasingly relevant not only for mechanistic understanding, but also for improving prognostic models and identifying patient subsets that may benefit from specific therapeutic strategies [30,60].

### 3.2. The Non-Coding RNA Regulome

Non-coding RNAs (ncRNAs), including microRNAs (miRNAs), long non-coding RNAs (lncRNAs), and circular RNAs (circRNAs), regulate gene expression in CRC [61,62]. MiRNAs such as miR-21, miR-135b, and miR-200c modulate post-transcriptional silencing and affect proliferation, apoptosis, and metastasis. Serum miR-21 has also shown diagnostic potential [63]. Oncogenic lncRNAs such as HOTAIR, MALAT1, and CCAT1 promote metastasis through mechanisms including PRC2-mediated epigenetic silencing. CircRNAs such as circHIPK3 and circRNA_100290 function as miRNA sponges or protein scaffolds, contributing to chemoresistance [64].

Beyond their individual expression changes, non-coding RNAs in CRC increasingly appear to function within broader regulatory networks rather than as isolated molecules. In particular, competing endogenous RNA (ceRNA) mechanisms provide one framework for understanding how lncRNAs and circRNAs modulate gene expression by sequestering shared miRNAs. This may relieve repression of downstream mRNA targets. Through these interactions, ncRNA-mediated networks may influence key CRC-associated processes, including epithelial–mesenchymal transition, proliferation, invasion, stemness, immune signaling, and drug resistance. This is especially relevant in CRC, where transcript abundance alone may not fully capture effective regulatory output. A network-based view also helps explain why dysregulated miRNAs, lncRNAs, and circRNAs are increasingly being investigated not only as individual biomarkers, but also as functionally connected regulators of tumor behavior and therapeutic response [65,66]. For example, circRNA- and lncRNA-centered ceRNA circuits have been reported to regulate CRC growth, migration, and treatment resistance through miRNA-mediated control of oncogenic pathways [67].

### 3.3. Single-Cell and Spatial Transcriptomics

Single-cell RNA-seq (scRNA-seq) resolves tumor microenvironment heterogeneity. It can identify functionally distinct tumor-associated fibroblast subsets that promote progression and reveal therapeutic targets [68]. Spatial transcriptomics preserves tissue architecture while profiling gene expression. It can map expression patterns to four anatomical regions: tumor core, stromal interface, immune infiltration zones, and normal epithelium [69]. These analyses show TMSB4X enrichment in tumor regions, suggesting a role in tumorigenesis. They also reveal active stromal-tumor interface communication via ligand-receptor pairs such as C5AR1-RPS19. In addition, they identify region-specific immune infiltration patterns, with immune-excluded phenotypes exhibiting T cell separation by stromal barriers [70].

## 4. Proteomics: The Functional Effector Layer

Proteomics provides essential functional insights in CRC by directly linking genotype to phenotype. As proteins execute cellular functions and serve as therapeutic targets, proteomic profiling elucidates disease mechanisms, reveals biomarkers, and identifies actionable targets [71]. Recent mass spectrometry advances have greatly improved the depth and coverage of proteomic analysis. These advances enable comprehensive characterization of protein expression, post-translational modifications, and protein–protein interactions in CRC tissues and biofluids [72]. Spatial transcriptomics has particular value in colorectal cancer because it allows transcriptional programs to be interpreted within the architectural context of the tumor microenvironment rather than in dissociated cell populations alone. This is especially important in CRC, where stromal activation, immune-cell localization, and epithelial–mesenchymal interactions can vary substantially across tumor regions [73]. By preserving spatial information, these approaches can distinguish tumor core, invasive front, stromal-rich niches, and immune-excluded versus immune-infiltrated areas, thereby providing a more precise view of local tumor ecology [74]. Spatial transcriptomic analyses have begun to uncover ligand–receptor interactions and region-specific communication among malignant, stromal, endothelial, and immune cells. This spatial information adds important biological context that is not captured by bulk profiling alone. It may help explain differences in progression, metastasis, and treatment response among tumors with otherwise similar molecular features [75]. In this sense, spatial transcriptomics is not only a high-resolution mapping tool. It also serves as an important bridge between molecular subtype classification and the biological organization of the CRC tumor microenvironment. Accordingly, spatial transcriptomics provides a way to link molecular heterogeneity with clinically relevant microenvironmental states. These include immune exclusion, stromal remodeling, and region-specific signaling programs that may influence prognosis and therapeutic sensitivity.

### 4.1. Technological Advances

Mass spectrometry-based proteomics has revolutionized CRC molecular characterization. Liquid chromatography-tandem mass spectrometry (LC-MS/MS) achieves proteome coverage exceeding 10,000 proteins from single biopsies with high sensitivity and specificity. Quantitative approaches include Tandem Mass Tag (TMT) isobaric labeling and Data-Independent Acquisition (DIA). TMT enables multiplexed analysis of up to 18 samples in a single run. By contrast, DIA supports cost-effective and reproducible workflows in large cohorts with coefficient of variation below 20% [76]. Targeted phosphoproteomics reveals dysregulated signaling including β-catenin (WNT) and AKT (PI3K/AKT) phosphorylation, identifying therapeutic vulnerabilities [77]. Glycoproteomics characterizes altered glycosylation patterns affecting adhesion, invasion, and immune recognition [78].

Emerging spatial proteomics technologies, including imaging mass cytometry (IMC) and multiplexed ion beam imaging (MIBI), have expanded the scope of proteomic analysis. These approaches enable spatially resolved, single-cell protein quantification within intact tissues. They also allow mapping expression across tumor, stromal, and immune compartments [79]. These approaches reveal spatial organization and cell–cell interactions, identifying distinct proteomic signatures at tumor invasion fronts and immune exclusion zones.

Different proteomic platforms offer complementary rather than interchangeable advantages in CRC research. Conventional LC-MS/MS provides broad proteome coverage and is well suited for discovery-oriented studies, but its performance can be influenced by sample complexity, instrument time, and batch effects [80]. TMT-based workflows enable high multiplexing and are advantageous for comparative analyses across multiple samples. However, ratio compression and dependence on careful batch design may affect quantitative accuracy [81]. DIA approaches generally offer better reproducibility and scalability for large cohorts. This makes them attractive for biomarker verification studies. Nevertheless, depending on the spectral library and acquisition settings, they may provide lower depth for some low-abundance proteins. Targeted phosphoproteomics is particularly useful for interrogating pathway activation and drug-response signaling [82]. By contrast, spatial proteomics platforms such as IMC and MIBI provide critical contextual information by resolving protein expression within tumor, stromal, and immune niches. However, these approaches are associated with higher cost, lower throughput, and greater analytical complexity. Accordingly, platform selection should be guided by the specific clinical or biological question. This may include biomarker discovery, pathway interrogation, cohort-scale validation, or spatial characterization of the tumor microenvironment [83]. The main applications are summarized in Table 5.

### 4.2. Proteomic Biomarkers and Pathways

Proteomic profiling has identified clinically relevant biomarkers across the diagnostic-prognostic-predictive spectrum [86]. Compared with traditional serum markers such as CEA and CA19-9, several emerging protein biomarkers show improved diagnostic sensitivity for colorectal cancer detection. These biomarkers include S100A9, MMP9, and Annexin A2 (Table 6). Proteomic validation studies have further demonstrated that multi-marker panels incorporating these candidates can achieve AUC values greater than 0.90, significantly improving early-stage detection accuracy [87]. Prognostic tissue signatures include mesenchymal proteins (vimentin, N-cadherin) correlating with poor outcomes, whereas epithelial markers (E-cadherin, cytokeratins) associate with favorable prognosis [88]. Proteins including galectin-3, cathepsin D, and S100A4 stratify patients into distinct risk categories beyond conventional staging [89]. For therapeutic targeting, membrane receptor quantification (EGFR, VEGFA, MET, HER2) and immune checkpoint profiling (PD-L1, PD-L2, LAG-3) guide treatment selection [89]. Critically, 30–40% of genes show poor mRNA-protein correlation, emphasizing proteomics’ superior functional assessment. This discordance has been quantitatively demonstrated in CPTAC colorectal cancer studies. In the CPTAC proteogenomic analysis of colon and rectal cancer, the mean Spearman correlation between steady-state mRNA and protein abundance across individual samples was 0.47. However, when mRNA and protein variation were compared across tumors, the mean correlation dropped to 0.23, and only 32% of mRNA–protein pairs showed statistically significant correlation. These findings indicate that transcript abundance alone is often insufficient to predict functional protein output in CRC and support the integration of proteomics into multi-omics stratification frameworks [90]. A representative example is provided by the Clinical Proteomic Tumor Analysis Consortium (CPTAC) proteogenomic study in colorectal cancer. This study showed that protein activity often does not correlate directly with mRNA abundance. This was particularly evident for post-translational modifications. such as phosphorylation. CPTAC analyses revealed that activation of key oncogenic pathways, including the WNT/β-catenin and PI3K/AKT signaling axes, is frequently driven by phosphorylation dynamics rather than transcriptional upregulation. For instance, signaling intermediates such as AKT and GSK3β may exhibit stable mRNA expression levels. However, their phosphorylation states can be markedly increased. This increase may enhance downstream signaling activity and promote tumor proliferation, survival, and immune evasion [91]. These findings underscore that phosphoproteomic profiling can capture functional pathway activation more accurately than transcriptomic data alone, highlighting the critical role of post-translational regulation in colorectal cancer biology. Phosphoproteomics further adds clinically relevant information by identifying targetable signaling states that may not be evident at the DNA or mRNA level. In CPTAC analyses of colon cancer, MSI-H tumors with reduced CD8 T-cell infiltration showed increased glycolytic activity.

This finding suggests that glycolytic rewiring may represent a potential strategy to overcome resistance to immune checkpoint blockade. In addition, integrated phosphoproteomic analysis of metastatic CRC showed that kinase-substrate network features could distinguish metastatic behavior. These features were also associated with response to kinase inhibitors, including afatinib, gefitinib, and regorafenib. Together, these findings highlight the translational value of phosphoproteomics for treatment stratification [10,92].

**Table 6:** Representative protein biomarkers and therapeutic targets in CRC. Representative proteins from CRC proteomics with biological roles and clinical applications. Established markers (CEA) enable monitoring; novel candidates (S100A9, Annexin A2) show diagnostic potential; therapeutic targets (VEGF-A, PD-L1) demonstrate clinical translation.

Protein	Specimen Source	Main Biological Role	Potential Clinical Application	Translational Status	Ref.
CEA	Serum/plasma	Traditional CRC-associated marker with limited specificity	Used for surveillance and recurrence monitoring	Established clinical use	[93]
S100A9	Tissue/serum	Inflammatory and tumor-associated protein linked to early disease and immune modulation	Candidate diagnostic biomarker; may improve early detection in combination panels	Investigational	[94]
MMP9	Tissue/serum	Involved in extracellular matrix degradation and invasion	Candidate prognostic biomarker for invasion and metastasis	Investigational	[95]
Vimentin	Tissue/serum	EMT-associated protein linked to invasion and metastatic potential	Indicator of aggressive phenotype and tumor progression	Investigational	[96]
Cathepsin D	Tumor tissue/serum	Protease associated with progression, recurrence, and poor outcome	Candidate prognostic biomarker with possible therapeutic relevance	Investigational	[97]
PD-L1	Tumor tissue	Immune checkpoint ligand involved in immune evasion	Predictive biomarker context for immunotherapy; therapeutic target	Clinically relevant in selected settings	[98]
VEGF-A	Tumor tissue/serum	Central mediator of angiogenesis	Therapeutic target for anti-angiogenic treatment	Established therapeutic relevance	[99]
Annexin A2	Plasma/exosomes/tissue	Membrane- and exosome-associated protein involved in invasion and progression	Candidate liquid biopsy biomarker and non-invasive monitoring marker	Investigational	[100]

Abbreviations: EMT, epithelial–mesenchymal transition; CEA, carcinoembryonic antigen; PD-L1, programmed death-ligand 1; VEGF-A, vascular endothelial growth factor A.

Despite the rapid expansion of proteomic biomarker discovery for colorectal cancer diagnosis, prognosis, and treatment stratification, clinical translation remains limited [101]. Pre-analytical variability, including differences in tissue handling, ischemia time, storage conditions, and biofluid processing, can substantially affect measured protein abundance [102]. In addition, cross-platform heterogeneity, differences in peptide identification pipelines, and limited inter-laboratory standardization hinder reproducibility and complicate comparisons across studies. Proteomic assays also face several intrinsic challenges. These include the wide dynamic range of the human proteome, the low abundance of clinically relevant signaling proteins, and the context dependence of post-translational modifications. Moreover, many candidate biomarkers have been derived from relatively small retrospective cohorts and still lack independent external validation or prospective clinical testing. Cost, turnaround time, infrastructure requirements, and the need for specialized bioinformatics support further restrict implementation in routine pathology and oncology settings. Therefore, the most promising path toward clinical application may lie in rigorously validated multi-protein panels. These panels should be integrated with genomic, transcriptomic, and clinicopathologic features. Such an approach may improve diagnosis, risk stratification, and disease monitoring [103].

**Figure 4:**
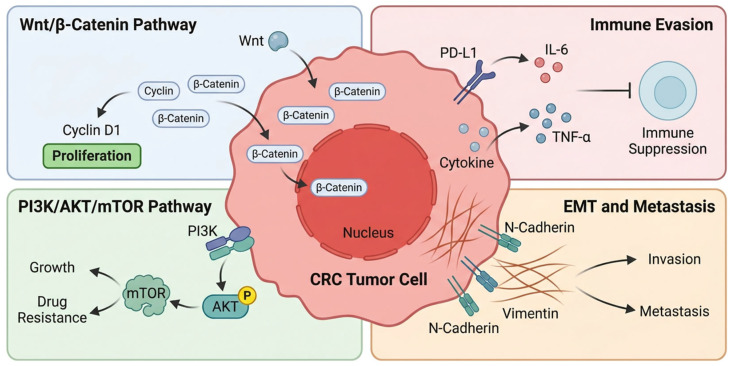
Dysregulated signaling pathways in CRC. Key altered pathways: WNT/β-catenin, PI3K/AKT/mTOR, epithelial–mesenchymal transition (EMT), and immune evasion. Aberrant β-catenin and Cyclin D1 drive proliferation; phosphorylated AKT and mTOR enhance survival and resistance; EMT markers (vimentin, N-cadherin) promote metastasis; immune-suppressive proteins (PD-L1, IL-6, TNF-α) enable immune evasion and support immunotherapy rationale.

Comparative proteomics reveals resistance mechanisms including alternative pathway activation (MET, AXL) mediating EGFR inhibitor resistance, and upregulated drug efflux pumps (ABCB1, ABCG2) conferring chemotherapy resistance (Figure 4) [104]. Tumor-derived exosomes carrying HSP70, TGF-β1, and Annexin A2 enable non-invasive disease monitoring, treatment response assessment, and minimal residual disease detection [105]. Pathway-level analyses reveal dysregulated networks including aberrant β-catenin (WNT), phosphorylated AKT (PI3K/AKT/mTOR), immune-suppressive proteins (PD-L1, IL-6), and EMT mediators (vimentin, N-cadherin) driving invasion and therapy resistance [106].

### 4.3. Translational Outlook

As proteomics matures, it increasingly informs personalized oncology in CRC. Proteogenomics integrates proteomic data with genomic and transcriptomic information. It has enabled the construction of comprehensive molecular atlases and may support individualized therapeutic interventions. Furthermore, proteomic biomarkers are being incorporated into early-phase clinical trials as companion diagnostics or predictive indicators, accelerating the clinical translation of multi-omics researcher. Overall, the translational value of proteomics in CRC depends not only on biomarker discovery. It also depends on platform-appropriate study design, analytical standardization, and prospective validation in clinically representative cohorts.

## 5. Metabolomics: The Phenotypic Signature of Reprogramming

Metabolomics, the comprehensive analysis of small-molecule metabolites, provides a dynamic snapshot of tumor physiology reflecting integrated genetic, transcriptomic, proteomic, and environmental effects. In CRC, metabolomic profiling has yielded critical insights into tumor metabolism, biomarker discovery, and disease progression mechanisms.

### 5.1. Analytical Technologies and Metabolic Hallmarks

Metabolomic investigations rely on several high-throughput analytical platforms. Nuclear magnetic resonance (NMR) spectroscopy is widely used for reproducible biofluid analysis. Mass spectrometry coupled with liquid or gas chromatography (LC-MS, GC-MS) enables sensitive broad-spectrum detection. Capillary electrophoresis-MS (CE-MS) is particularly useful for resolving polar metabolites, including organic acids and bile salts [107]. Both targeted and untargeted approaches are applied to tissue biopsies, serum/plasma, stool, and urine, enabling invasive and non-invasive disease monitoring.

CRC cells exhibit profound metabolic reprogramming across multiple pathways. Aerobic glycolysis (Warburg effect) drives preferential glucose-to-lactate conversion under normoxia, with upregulated lactate dehydrogenase A (LDHA) and accumulated lactate and pyruvate [108]. Importantly, metabolic reprogramming in CRC is not uniform across molecular or clinicopathologic subtypes. Metabolomic studies indicate that CRC can be stratified into metabolically distinct subgroups with differences in lipid, nucleotide, and carbohydrate metabolism, suggesting that tumor behavior and prognosis may be influenced by subtype-specific metabolic dependencies. In addition, metabolic phenotypes vary according to genetic context and tumor location. For example, experimental metabolomic profiling of genetically engineered intestinal tumors has shown that common driver alterations such as APC, KRAS, and TP53 generate distinct metabolic states rather than a single shared CRC metabolic program. Likewise, right-sided and left-sided CRCs exhibit partially divergent metabolomic landscapes, further supporting the existence of clinically relevant metabolic heterogeneity in CRC [109]. Glutamine serves as an anaplerotic TCA cycle substrate under hypoxia, generating altered α-ketoglutarate, succinate, and fumarate levels. Enhanced lipogenesis produces abnormal phosphatidylcholines, sphingolipids, lysophosphatidylcholine (LPC), and ceramides supporting membrane synthesis and oncogenic signaling [110]. Dysregulated amino acid metabolism—particularly arginine, tryptophan (kynurenine pathway), methionine, and branched-chain amino acids—promotes proliferation and immune evasion.

### 5.2. Microbial Metabolites and the Gut-Tumor Axis

**Figure 5:**
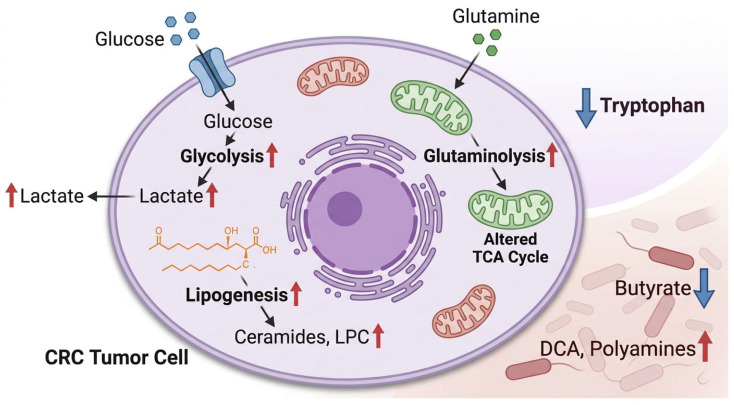
Metabolic reprogramming in CRC. CRC cells exhibit enhanced glycolysis, glutaminolysis, and lipogenesis with elevated lactate, ceramides, and lysophosphatidylcholine (LPC). Depleted tryptophan and butyrate alongside enriched microbial metabolites (deoxycholic acid, polyamines) drive tumor growth, immune evasion, and inflammation, representing diagnostic biomarkers and therapeutic targets.

The gut microbiota influences CRC pathogenesis through metabolites with opposing properties. Short-chain fatty acids (SCFAs), including butyrate, acetate, and propionate, are produced through dietary fiber fermentation. These metabolites exhibit anti-tumor effects through histone deacetylase (HDAC) inhibition. They can promote cell cycle arrest, apoptosis, and differentiation, and reduced SCFA levels have been associated with CRC risk [111]. Conversely, secondary bile acids (deoxycholic acid, lithocholic acid) generated by bacterial 7α-dehydroxylation accumulate in high-fat, low-fiber diets, inducing DNA damage and inflammatory signaling via NF-κB and STAT3 activation [112]. Polyamines, including putrescine, spermidine, spermine, become dysregulated in CRC. This dysregulation can promote tumor growth and immunosuppression. Hydrogen sulfide (H_2_S), by contrast, shows dose-dependent effects. Low concentrations support epithelial barrier function, whereas high levels induce genotoxicity [113]. These findings highlight the critical role of diet-microbiota-metabolite interactions in CRC development, presenting opportunities for microbiome-targeted interventions (Figure 5). These metabolic interactions may be especially relevant in early-onset colorectal cancer (EOCRC). Although the metabolic biology of EOCRC is still being defined, emerging evidence suggests that younger-onset disease may not simply mirror the metabolic features of later-onset CRC. Recent integrative metagenomic-metabolomic studies have identified distinct gut microbiome-derived phenotypes in EOCRC. These findings suggest that altered bile acid handling, short-chain fatty acid imbalance, and broader changes in amino acid and lipid metabolism may contribute to tumor initiation in younger individuals. This is consistent with the view that diet, obesity, antibiotic exposure, and other early-life influences may reshape the intestinal metabolic environment long before overt malignancy develops. For this reason, EOCRC represents a particularly informative setting in which metabolomics and microbiome profiling can be studied together rather than in isolation.

In addition, metabolic flux analysis and stable-isotope tracing provide functional insight beyond steady-state metabolite abundance by revealing nutrient utilization and pathway activity. In CRC, isotope-tracing studies have shown that oncogenic PIK3CA mutations reprogram glutamine metabolism, highlighting how flux-based approaches can uncover targetable metabolic dependencies [114,115].

Importantly, the microbiome–metabolite axis in CRC is supported not only by associative studies but also by functional evidence. For example, in gnotobiotic mouse models, a high-fiber diet combined with butyrate-producing bacteria increased luminal butyrate levels. This was accompanied by a reduced tumor burden, supporting a causal tumor-suppressive role for this microbial metabolite [6]. Conversely, paired microbiome–metabolome analyses in CRC-susceptible mice showed that high-fat diet–driven microbial shifts increased bile acid alterations, including microbially modified bile acid species, and promoted disease progression. These findings indicate that selected microbiota-derived metabolites can act as functional mediators of tumor promotion or suppression rather than merely correlating with disease status [116].

### 5.3. Translational Potential

Metabolomics has emerged as a powerful tool for CRC precision oncology (Table 7), with serum or stool metabolomic signatures [117]. Several metabolite panels have shown promising diagnostic performance in CRC, but these estimates vary by biospecimen type, cohort design, and validation strategy. For example, one serum metabolomics study reported an 8-metabolite panel with an AUC of 0.968, sensitivity of 95%, and specificity of 100% in CRC diagnosis, whereas another integrative plasma metabolomics study identified a 17-metabolite panel that achieved AUCs ranging from 0.848 to 0.987 across discovery and three validation cohorts. Accordingly, statements regarding diagnostic sensitivity should be linked to specific studies rather than generalized across all metabolite panels [117,118]. Prognostically, altered levels of sarcosine, choline derivatives, and succinate correlate with tumor stage, lymph node involvement, and recurrence risk. Longitudinal profiling can also be used to monitor treatment response monitoring. For example, lactate levels may decrease after treatment, whereas SCFAs levels may increase [119]. Additionally, metabolomics guides personalized interventions by evaluating dietary impacts (fiber intake) and probiotic/prebiotic supplementation effects, informing nutrition-based therapeutic strategies.

## 6. Microbiomics: The Tumor Ecosystem Modulator

The gut microbiome critically influences CRC pathogenesis through genotoxic insult, chronic inflammation, metabolic reprogramming, and immune modulation. Multi-omics integration combining metagenomics, metatranscriptomics, and metabolomics has revealed specific microbial signatures associated with tumor initiation, progression, and therapeutic response.

### 6.1. Dysbiosis and Key Pathobionts

CRC exhibits distinct gut dysbiosis characterized by depletion of beneficial commensals (e.g., *Faecalibacterium*, *Roseburia*) and enrichment of pro-carcinogenic pathobionts [129]. *Fusobacterium nucleatum* promotes tumor progression via FadA/E-cadherin signaling and immune suppression via Fap2/TIGIT interaction; a candidate diagnostic and prognostic biomarker [130]. Mechanistic studies support causal roles for several CRC-associated pathobionts rather than simple enrichment alone. In particular, *Fusobacterium nucleatum* has been shown to promote colorectal tumorigenesis through its FadA adhesin. FadA binds to E-cadherin and activates β-catenin signaling. This, in turn, enhances epithelial proliferation and oncogenic transcriptional programs [131]. In parallel, the Fap2 virulence factor can bind TIGIT on immune cells and contribute to immune evasion within the tumor microenvironment. Enterotoxigenic *Bacteroides fragilis* (ETBF) exerts tumor-promoting effects through the *B. fragilis* toxin (BFT). This toxin disrupts epithelial integrity and activates STAT3 signaling. In preclinical models, it also drives IL-17/Th17-predominant inflammation [132]. Likewise, pks+ *Escherichia coli* has been shown to induce colibactin-mediated DNA damage. Organoid and genomic studies have linked this exposure to characteristic SBS-pks and ID-pks mutational signatures. These findings support a direct role for pks+ *E. coli* in early colorectal tumorigenesis [133]. Where prevalence estimates are provided, these should be interpreted as study-specific rather than universal values. For example, one early clinical study detected ETBF in 38% of stool isolates from CRC patients, compared with 12% in controls. More recent tumor-based work also reported that a CRC-associated *F. nucleatum* clade was present in approximately half of colorectal tumor samples [134]. Enterotoxigenic *Bacteroides fragilis* (ETBF) secretes a toxin (BFT) that cleaves E-cadherin, activating oncogenic signaling and promoting a pro-inflammatory Th17 response [135]. Polyketide synthase-positive *Escherichia coli* (pks+ *E. coli*) produces the genotoxin colibactin. This toxin induces DNA double-strand breaks and is associated with characteristic mutational signatures, including SBS-pks, ID-pks. Its higher abundance in early-stage tumors suggests that this pathobiont may contribute to tumor initiation and early-onset CRC [136]. These microbiome-related mechanisms may be especially relevant in EOCRC. In contrast to the traditional view that younger patients simply develop CRC earlier, accumulating evidence suggests that EOCRC may arise in a somewhat different ecological context, shaped by altered microbial communities, diet-related exposures, and microbe-derived metabolites. This possibility is supported by recent work describing distinct gut-microbiome-derived phenotypes in EOCRC, as well as by observations linking pathobionts such as pks+ *E. coli* to early-stage tumorigenesis and younger-age disease. Taken together, these findings suggest that microbial dysbiosis in EOCRC is not only a candidate biomarker, but may also contribute to a biologically shifted route of carcinogenesis.

### 6.2. Translational Applications and Therapeutic Modulation

Microbiome profiling demonstrates significant clinical potential (Table 8). Fecal signatures, particularly *F. nucleatum*, ETBF, and pks+ *E. coli* enrichment, show diagnostic accuracy comparable to fecal immunochemical testing, with integrated SCFA and bile acid profiles enhancing early detection [135,136,137]. Prognostically, tissue-resident microbial risk scores predict outcomes independently of established parameters, with high *F. nucleatum* burden correlating with shorter disease-free survival, increased recurrence, and chemotherapy resistance [138]. Therapeutic strategies include dietary interventions promoting SCFA-producing bacteria and suppressing carcinogenic taxa. They also include probiotic supplementation with *Lactobacillus*, *Bifidobacterium*, *Faecalibacterium*. Other approaches include targeted pathogen elimination using narrow-spectrum antibiotics or bacteriophages, as well as fecal microbiota transplantation (FMT) [139]. Preclinical studies demonstrate that FMT from Fn-high donors confers immunotherapy sensitivity in mouse models. Baseline microbiome composition has also been associated with immune checkpoint inhibitor response. These findings suggest that microbiome profiling may help guide patient selection and inform rational combination strategies [140].

## 7. Integrative Multi-Omics Frameworks: From Data to Insight

**Figure 6:**
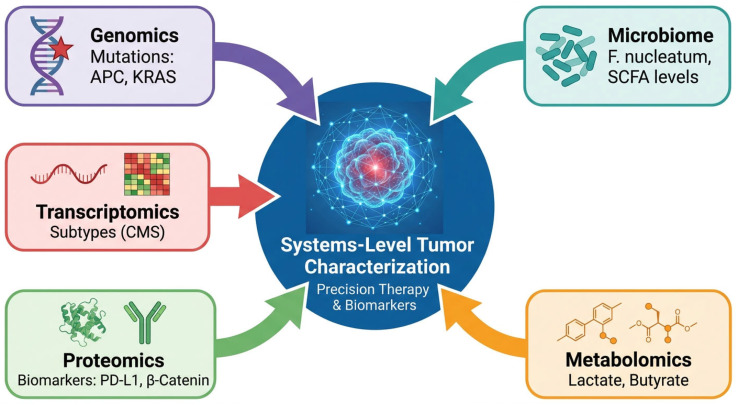
Integrative multi-omics framework in CRC. This schematic illustrates the convergence of genomic, transcriptomic, proteomic, metabolomic, and microbiome data into a unified systems-level understanding of CRC. Integration across these omics layers enhances tumor classification, biomarker discovery, and precision therapeutic strategies.

The true power of multi-omics in colorectal cancer research lies not in the parallel generation of disparate datasets, but in their systematic integration (Figure 6). This synthesis moves beyond descriptive cataloguing to reveal the causal, systems-level mechanisms that drive tumor biology, therapeutic response, and resistance. By correlating variations across molecular layers, integration transforms heterogeneous data into a coherent model of disease. This approach can identify master regulatory networks and context-specific vulnerabilities. These features may not be detectable with any single-omics approach.

### 7.1. Artificial Intelligence Methods for Multi-Omics Integration in Colorectal Cancer

Computational approaches, including artificial intelligence (AI), have become increasingly important in CRC multi-omics research. This is because genomic, transcriptomic, proteomic, metabolomic, and microbiome datasets differ substantially in scale, sparsity, and biological interpretation. When these layers are analyzed separately, important cross-omics relationships may be missed. Integrative computational approaches help address this problem. They provide a practical way to better capture molecular complexity and support subtype classification, biomarker discovery, prognosis assessment, and treatment-response prediction [145,146]. Several established algorithms are particularly relevant in this setting. Multi-Omics Factor Analysis (MOFA) is an unsupervised latent factor framework for identifying shared and omics-specific sources of variation across omics layers [147]. In CRC, MOFA-based integration has been applied to CPTAC-derived multi-omics data and has identified prognostically relevant latent factors associated with stromal and extracellular matrix-related programs [148]. iCluster performs joint clustering of heterogeneous molecular data and is useful for subtype discovery [149]. Data Integration Analysis for Biomarker discovery using Latent cOmponents (DIABLO) supports supervised biomarker selection across omics layers [150], whereas similarity network fusion (SNF) integrates sample-level similarity networks to improve patient stratification [151]. Together, these methods provide complementary strategies for integrating transcriptomic, proteomic, metabolomic, and microbiome-related data in CRC.

Deep learning and network-based approaches have further expanded the analytical toolbox for CRC multi-omics integration. Autoencoders and variational autoencoders can reduce dimensionality while preserving informative latent structure, and they have been explored in CRC for patient stratification, survival prediction, and identification of hidden molecular subgroups [152]. Graph neural networks (GNNs) are also promising because they preserve biological context and model interactions across multiple omics layers [153]. In cancer research more broadly, methods such as Multi-Omics Graph cOnvolutional NETworks (MOGONET) have shown that graph-based integration can improve patient classification and biomarker identification. These findings suggest strong potential for pathway-level inference, tumor-microenvironment analysis, and host-microbiome interaction modeling in CRC [154]. In parallel, transfer learning may help address the practical limitation of small CRC multi-omics cohorts by adapting models pretrained on large public resources such as the Cancer Genome Atlas (TCGA) and the Clinical Proteomic Tumor Analysis Consortium (CPTAC) [9,90]. However, clinical translation requires not only predictive performance but also interpretability. Explainable AI methods such as SHAP and LIME can help identify the features driving model predictions and improve biological and clinical understanding [155]. Overall, the future value of AI in CRC multi-omics will depend on selecting appropriate analytical frameworks, using adequately validated datasets, and combining computational inference with careful experimental and clinical interpretation. Despite the growing power of multi-omics integration methods, several technical challenges remain. Batch effects are a major concern because datasets are often generated across different laboratories, platforms, time points, and preprocessing pipelines. If not adequately corrected, these effects can obscure true biological signals and reduce reproducibility [156]. Data normalization is also critical, as each omics layer has distinct distributions, dynamic ranges, and missing-data patterns, making direct comparison difficult. In addition, cross-platform variability may limit the transferability of models and complicate the integration of public and locally generated datasets [145,146,157]. These issues are particularly relevant in CRC, where multi-omics cohorts are often assembled from heterogeneous sources. Accordingly, robust preprocessing, harmonization, and external validation are essential before integrated models can be reliably translated into clinical research or practice.

### 7.2. Flagship Integrative Studies

Large-scale consortia have been instrumental in generating foundational multi-omics resources for CRC (Table 9). TCGA provided a cornerstone with its integrated genomic and transcriptomic characterization. Building upon this, the CPTAC delivered a seminal proteogenomic atlas of colorectal cancer, integrating whole-exome sequencing, RNA sequencing, global proteomics, and phosphoproteomics [158]. This study yielded several important insights. Proteomic data often provided more accurate prognostic stratification than transcriptomic data alone. The study also identified key post-translational drivers, such as specific phosphorylation events, that activate oncogenic pathways independent of mRNA abundance [159]. Such projects provide the essential datasets and proof-of-concept that multi-omics integration can reveal biology masked by single-layer analyses.

### 7.3. Conceptual Advancements from Integration

The application of integrative frameworks has led to several key conceptual advances in understanding CRC. First, it is resolving heterogeneity within established molecular classifications. For instance, proteogenomic analyses have begun to deconvolute the Consensus Molecular Subtype 4 (CMS4). These studies have identified “immune-hot” and “immune-cold” subsets with markedly different microenvironments. Despite similar transcriptional signatures, these subsets may show different responses to immunotherapy [164]. Second, integration is elucidating the multifaceted nature of therapy resistance. It shows that resistance is rarely caused by a single event. Instead, it usually reflects coordinated adaptation across multiple molecular layers. This may include the emergence of secondary genomic mutations, such as KRAS amplification, transcriptional rewiring of bypass pathways, proteomic switching of receptor tyrosine kinases (e.g., MET upregulation), and metabolic flexibility in fuel utilization [165]. Finally, integration is decoding the dynamic dialogue between the host and its microbiome. By combining metagenomics, metabolomics, and host transcriptomics, these studies clarify how specific pathobionts such as *Fusobacterium nucleatum* locally modulate the tumor immune microenvironment. For example, *F. nucleatum* may deplete butyrate and recruit immunosuppressive cells. These effects can influence carcinogenesis and treatment sensitivity [166]. These advancements highlight that a holistic, multi-layered perspective is indispensable for understanding the complex biology of CRC and translating discoveries into effective precision medicine.

### 7.4. Multi-Omics Reveals Hierarchical Regulation of CRC Biology

The occurrence and development of colorectal cancer are not dominated by changes at a single molecular level. Instead, they arise from a hierarchical regulatory system that includes genomic potential, transcriptional regulation, protein function, and metabolic phenotypes [167]. Multi-omics integrative studies have begun to reveal this layered structure, indicating that tumor behavior is not determined by a single mutation but is shaped by cross-layer regulatory coherence. At the genomic level, driver gene mutations such as APC, KRAS, and TP53 lay the groundwork for the malignant potential of tumor [168]. However, these changes alone do not determine pathway activity or tumor phenotype. Transcriptomic data introduce a regulatory dimension, reflecting how tumor cells adjust their gene expression programme in response to intrinsic gene mutations and external environmental pressures. Yet, transcriptional states do not necessarily translate into functional activation. Proteomic and phosphoproteomic studies indicate that numerous oncogenic pathways, including WNT and PI3K/AKT, are activated through post-translational modifications. These include phosphorylation rather than transcriptional upregulation. For instance, even when mRNA levels remain stable, phosphorylation of key signaling molecules like AKT or GSK3β can amplify downstream signals. Therefore, this functional level represents the true signaling state of the tumor, linking genetic potential with biological execution. Beyond intracellular signal transduction, metabolomic analysis captures the phenotypic output of these regulatory networks. Alterations in glycolysis, lipid metabolism, and amino acid metabolic pathways reflect the downstream consequences of integrated pathway activation, connecting molecular events with tumor growth, immune evasion, and therapy resistance. Finally, the microbiome introduces an external regulatory layer, reshaping tumor biology through metabolite-driven immune and inflammatory signaling. Microbial communities do not act directly as oncogenic drivers but influence host signal transduction and metabolic states, thereby participating in the regulation of the tumor ecosystem.

### 7.5. From Correlation to Causation in Multi-Omics CRC Research

Although multi-omics integration has greatly facilitated the discovery of molecular associations in CRC, an important challenge remains. It is still difficult to move from correlation-based observations to true mechanistic understanding. Many integrated datasets reveal co-occurring changes at the genomic, transcriptomic, proteomic, and metabolomic levels. However, these relationships do not necessarily indicate causal regulatory interactions. Recent advances are beginning to address this limitation.

Functional validation platforms are indispensable for transforming multi-omics correlations into mechanistic and causal insights. Patient-derived organoids (PDOs) provide physiologically relevant models that preserve tumor heterogeneity and enable pathway-level hypothesis testing, such as validating TGF-β inhibition in CMS4 colorectal cancer [169]. Complementarily, genome-wide CRISPR-Cas9 screening allows systematic interrogation of candidate drivers derived from integrative analyses and has uncovered synthetic lethal vulnerabilities in KRAS-mutant CRC [170].

Beyond genetic validation, spatial multi-omics approaches can provide additional mechanistic insight. These methods, including imaging mass cytometry and spatial transcriptomics, retain tissue architecture and enable causal mapping of intercellular signaling [171]. Notably, spatial profiling has demonstrated that TGF-β signaling in CMS4 tumors primarily originates from cancer-associated fibroblasts rather than malignant epithelial cells. At the translational level, pharmacotranscriptomics integrates drug-response phenotypes with multi-omics signatures to identify predictive biomarkers of sensitivity and resistance [172]. Large-scale pharmacogenomic resources such as GDSC and CTRP have thus facilitated therapy stratification based on molecular dependencies. Collectively, these platforms establish a validation continuum that bridges molecular discovery with functional causation and precision therapeutic prediction. For instance, perturbation of candidate regulatory factors identified from integrated datasets has shown that pathway remodeling and therapy resistance often arise from coordinated adaptive changes across multiple molecular layers. They are therefore rarely driven by a single genetic event. Spatial multi-omics further enhances causal inference by preserving tissue structure and enabling mapping of signaling interactions within the tumor microenvironment. These methods show that stromal cells and immune components actively contribute to pathway activation. This can occur through mechanisms such as TGF-β-mediated fibroblast signaling or cytokine-driven immune modulation. As a result, tumor behavior may be influenced independently of intrinsic tumor cell mutations.

Moreover, emerging computational frameworks that integrate causal network modelling and machine learning are aiding in the inference of regulatory hierarchies from complex datasets. By combining perturbation data with observational multi-omics maps, these approaches are moving from descriptive associations towards predictive and mechanistic insights. In conclusion, these advances suggest that the next frontier of multi-omics research is not limited to identifying molecular features. It also involves understanding how cross-layer interactions drive tumor evolution, immune evasion, and therapy resistance. Bridging the gap between correlation and causation will be essential for translating multi-omics findings into clinically applicable strategies in precision oncology.

## 8. Translational Applications and Clinical Integration

Multi-omics research is transforming clinical practice by driving advanced biomarker development, refining therapeutic decisions, and enabling dynamic disease monitoring for precision CRC oncology.

### 8.1. Biomarker Discovery and Validation

Multi-omics accelerates development of composite biomarkers capturing tumor complexity beyond single-analyte tests. Predictive power for immune checkpoint inhibitor response can be improved by integrating biomarkers from multiple omics layers. These include MSI-H status from genomics, cytotoxic T-cell infiltration signatures from transcriptomics, PD-L1 protein levels from proteomics, and immunostimulatory microbiome taxa from microbiomics. This multi-dimensional profiling explains variable MSI-H responses and identifies potential responders within microsatellite-stable populations, requiring prospective validation in large clinical trials.

### 8.2. Guiding Precision Therapy

Integration directly informs personalized strategies. Targeted therapy selection is guided by RAS/BRAF wild-type status for anti-EGFR antibodies, BRAF V600E for BRAF/EGFR inhibition, and HER2 amplification or NTRK fusions for kinase inhibitors [173]. For immunotherapy, gut microbiome composition modulates MSI-H/dMMR responses, with specific consortia predicting efficacy and representing therapeutic targets. Multi-omics analyses can reveal resistance mechanisms of treatment resistance. Examples include MET amplification during EGFR inhibition and PI3K upregulation with MAPK inhibition. These findings may help inform rational combination strategies in clinical trials.

### 8.3. Liquid Biopsy for Dynamic Monitoring

Multi-omics integration with liquid biopsy enables non-invasive, real-time disease management. Analyzing circulating tumor DNA (ctDNA), cell-free RNA (cfRNA), exosomal proteins, and plasma metabolites provides comprehensive molecular snapshots. This information may support minimal residual disease detection after surgery, molecular response monitoring, and early detection of resistance. Examples include KRAS mutations and MET amplification. These findings may help guide adaptive therapy [174].

### 8.4. Towards Clinical Implementation

Clinical translation requires a structured implementation. Baseline next-generation sequencing panels should assess genomic drivers, MSI status, and gene fusions. Deeper transcriptomic or proteomic profiling may be useful for atypical presentations or treatment resistance. Serial liquid biopsy may be particularly helpful for high-risk and metastatic patients. Integration of multi-omics data into electronic health records with clinical decision support tools generating patient-specific recommendations requires addressing data standardization, interoperability, clinician education, and cost-effectiveness.

Despite these advances, the routine clinical use of multi-omics in colorectal cancer remains difficult. Compared with conventional single-analyte assays, multi-omics tests involve several data layers and more complicated analytical workflows. This makes validation harder. Reproducibility needs to be shown across laboratories, instruments, sample-processing procedures, and bioinformatic pipelines [175]. These issues directly affect clinical adoption. They also make regulatory review more demanding. This is especially relevant for complex in vitro diagnostics and for models that include algorithm-based or AI-assisted interpretation [176,177]. In addition, many reported multi-omics signatures still come from retrospective datasets or single-center studies. Prospective multicenter validation remains limited [178]. Other barriers include the lack of standardized reference materials, inconsistent reporting practices, data-governance concerns, interoperability problems, and limited reimbursement pathways. For these reasons, future progress will depend not only on biomarker discovery. It will also require better standardization, clearer regulatory pathways, and stronger real-world clinical validation.

## 9. Challenges and Future Directions

**Figure 7:**
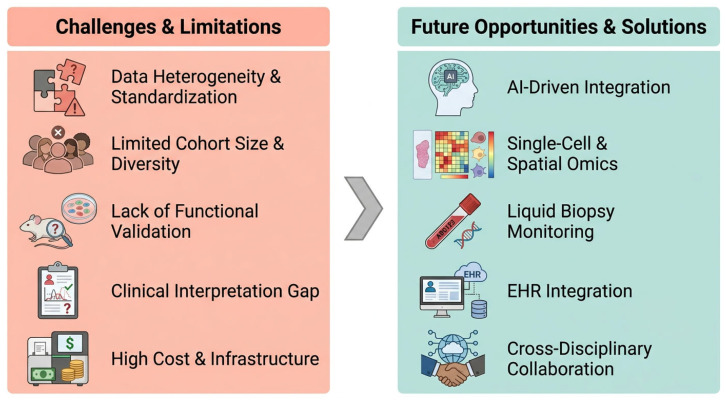
Challenges and future opportunities in multi-omics CRC research. This comparative schematic outlines key limitations of current multi-omics CRC research—including data standardization, sample diversity, validation gaps, and cost barriers—alongside emerging solutions such as AI integration, single-cell/spatial omics, liquid biopsy applications, and cross-disciplinary collaboration.

Despite transformative potential, multi-omics integration into routine CRC care faces significant technical, translational, and infrastructural hurdles that must be addressed to realize precision oncology (Figure 7).

### 9.1. Persistent Challenges

Technical and computational complexity remains a primary barrier. Lack of standardized protocols for sample collection, processing, and analysis across platforms introduces batch effects and technical noise confounding biological signals [179]. Robust, user-friendly bioinformatic pipelines for harmonizing heterogeneous data types remain critically needed, often limiting analysis to specialized groups. Most multi-omics-derived biomarkers have been identified in retrospective and underpowered cohorts. They therefore require prospective validation in large and diverse populations within clinical trials. Rigorous health-economic analyses are also needed to demonstrate cost-effectiveness. The high cost of advanced sequencing, mass spectrometry, and computational infrastructure risks creating a “genomic divide.” This could restrict the benefits of precision oncology benefits to well-resourced systems and further exacerbate global health disparities.

To ensure the equitable global implementation of multi-omics approaches, strategic priorities for resource-limited regions deserve attention. Firstly, a tiered diagnostic framework can be adopted, prioritizing targeted sequencing panels and key biomarker testing, which reduces technical barriers and costs as an alternative to whole-genome sequencing. Secondly, establishing regional biobanks can facilitate standardized sample collection and shared infrastructure, both improving data quality and avoiding redundant resource investment. Thirdly, bioinformatics platforms based on cloud computing provide scalable solutions for data processing, effectively reducing dependence on local high-end computing equipment. Finally, multi-omics tools can be integrated into existing screening systems, such as fecal immunochemical testing or colonoscopy referral pathways. This allows gradual implementation within established workflows and avoids the need to build separate frameworks. These strategies complement each other, offering a feasible path for cost-effective, stepwise implementation of precision oncology in resource-constrained settings. Importantly, these technical limitations are closely intertwined with regulatory and reimbursement barriers. Insufficient standardization, poor external reproducibility, and limited prospective evidence directly impede approval, clinical adoption, and payer coverage of multi-omics diagnostics.

### 9.2. Strategic Opportunities and Future Vision

Next-generation technologies will provide unprecedented resolution. These include single-cell multi-omics, which can simultaneously measure the transcriptome and epigenome, spatial molecular profiling, and real-time multi-analyte liquid biopsies. Together, these approaches may enable dynamic mapping of tumor evolution [180]. Artificial intelligence (AI) and machine learning are becoming indispensable for integrating complex datasets, building predictive treatment response models, and automating molecular report interpretation [181]. Progress requires global collaboration building large-scale, ethnically diverse biorepositories with longitudinal clinical annotation for generalizable discoveries. The field must advance through pragmatic clinical trials directly testing whether multi-omics-guided decisions improve patient outcomes versus standard pathways. Sustainable implementation demands investment in translational data scientist training and secure, cloud-based platforms democratizing advanced analytics access worldwide.

AI and machine learning are reshaping multi-omics integration by enabling hierarchical data interpretation, predictive modeling, and mechanistic insight generation. At the foundational level, deep learning approaches such as autoencoders and variational autoencoders (VAEs) facilitate dimensionality reduction by extracting latent representations from high-dimensional multi-omics datasets. These compressed features improve downstream tasks including patient stratification and survival prediction. Building upon this, graph-based learning methods provide a systems-level perspective [182]. Graph neural networks (GNNs) model multi-omics data as heterogeneous biological networks. In this framework, genes, proteins, and metabolites serve as nodes, while their functional interactions are represented as edges. This approach helps capture cross-layer relationships and may improve treatment-response prediction [183]. To address data scarcity in clinical cohorts, transfer learning enables knowledge reuse from large-scale public resources such as TCGA and CPTAC. By fine-tuning pre-trained models on smaller datasets, predictive performance can be enhanced in underpowered studies. Beyond predictive performance, explainable AI (XAI) techniques, including SHAP and LIME, provide interpretability by identifying key multi-omics features driving model outputs [184]. This facilitates biological insight generation and supports the formulation of experimentally testable hypotheses.

Despite these advances, challenges remain. Small sample sizes increase the risk of overfitting, standardized benchmarking frameworks are lacking, and external validation remains insufficient. Emerging federated learning strategies offer a potential solution by enabling multi-institutional model training. They allow models to be trained without sharing raw data, which helps preserve privacy while improving robustness and generalizability. Together, these AI-driven approaches provide a scalable framework for transforming high-dimensional multi-omics data into clinically actionable knowledge.

## 10. Conclusions

In conclusion, several points should be emphasized. First, colorectal cancer is highly heterogeneous, and no single omics approach can fully explain its complexity. Second, multi-omics provides a broader view of CRC biology by combining information from different molecular levels. This creates new opportunities for biomarker discovery, patient classification, and treatment selection. Third, important barriers still remain. These include technical variability, difficulties in data integration, limited standardization, and insufficient prospective validation. Clinical implementation is also restricted by cost, regulatory issues, and reimbursement challenges. Overall, multi-omics has strong potential in CRC research and precision oncology, but further standardization and real-world validation will be needed before it can be widely applied in routine practice.

## Figures and Tables

**Table 1:** Key signaling pathways and their genetic alterations in CRC. The table summarizes major dysregulated pathways, their frequently mutated genes, core biological functions, and supporting references.

Pathway	Key Genes Involved	Main Function	Ref.
Wnt signaling	APC, β-catenin	Regulates cell growth and differentiation	[16]
MAPK pathway	KRAS, BRAF, MEK	Controls cell proliferation and apoptosis	[17]
PI3K pathway	PIK3CA, PTEN	Involved in cell growth and survival signaling	[18]
TGF-β pathway	SMAD4	Regulates cell cycle and apoptosis	[19]
EGF pathway	EGFR, KRAS	Mediates cell signaling for growth and division	[20]

Abbreviations: APC, adenomatous polyposis coli; MAPK, mitogen-activated protein kinase; KRAS, Kirsten rat sarcoma viral oncogene homolog; BRAF, B-Raf proto-oncogene; MEK, MAPK/ERK kinase; PI3K, phosphatidylinositol 3-kinase; PIK3CA, phosphatidylinositol-4,5-bisphosphate 3-kinase catalytic subunit alpha; PTEN, phosphatase and tensin homolog; TGF-β, transforming growth factor beta; SMAD4, SMAD family member 4; EGF, epidermal growth factor; EGFR, epidermal growth factor receptor.

**Table 3:** Biological and clinical features of the consensus molecular subtypes in CRC. References: Summarized from [42,43], with additional CMS4-related stromal and immune microenvironment features adapted from [44].

Feature	CMS1 MSI Immune	CMS2 Canonical	CMS3 Metabolic	CMS4 Mesenchymal
Approximate prevalence	~14%	~37%	~13%	~23%
Predominant sidedness	More often right-sided	More often left-sided	Variable	Variable, often advanced-stage disease
Prognostic pattern	Poor survival after relapse; may benefit from immunotherapy	Best overall prognosis among CMS groups	Intermediate prognosis	Worse OS and RFS
Dominant biological features	Hypermutation, MSI, strong immune activation, frequent BRAF mutation, high CIMP	Epithelial differentiation, WNT and MYC activation, marked CIN/SCNA	Metabolic dysregulation, epithelial features, frequent KRAS mutation, lower CIN than CMS2	TGF-β activation, stromal invasion, angiogenesis, EMT, inflammatory and immunosuppressive microenvironment
Immune/stromal context	Diffuse immune infiltrate, TH1 and cytotoxic-cell rich	Relative immune quiescence, epithelial phenotype	Limited immune activation, metabolically driven phenotype	Stromal-rich, immune-excluded microenvironment
KRAS mutation frequency	~25%	~25%	~68%	~40%
BRAF mutation frequency	~40–45%	<1%	<10%	<10%

Abbreviations: CMS, Consensus Molecular Subtypes; MSI, microsatellite instability; CIMP, CpG island methylator phenotype; CIN, chromosomal instability; SCNA, somatic copy number alterations; EMT, epithelial–mesenchymal transition; TH1, T helper 1; OS, overall survival; RFS, relapse-free survival.

**Table 4:** Representative transcriptomic biomarkers and gene-expression signatures in CRC. Key coding and non-coding RNA biomarkers and gene-expression signatures, highlighting their functional roles, clinical relevance, and validation status in CRC.

Biomarker	Sample Source	Type	Main Function in CRC	Clinical Relevance	Validation Status	Ref.
miR-21	Tumor/plasma	miRNA	Promotes proliferation and invasion; diagnostic/prognostic biomarker	Non-invasive biomarker; detectable in blood	Investigational/emerging	[46,47]
HOTAIR	Tumor tissue	lncRNA	Enhances metastasis via PRC2-mediated gene silencing	Potential target for RNA-based therapy	Preclinical/exploratory	[48,49]
circHIPK3	Tumor tissue	circRNA	Acts as miRNA sponge; promotes chemoresistance	Emerging biomarker for drug resistance	Preclinical/exploratory	[50,51]
MMP7	Tumor tissue/serum	mRNA	Matrix remodeling and invasion; overexpressed in tumors	Diagnostic and prognostic marker	Investigational	[52,53]
Oncotype DX Colon	Tumor tissue	Gene Signature	Predicts recurrence risk and guides adjuvant chemotherapy	Used in clinical decision-making	Commercially available/clinically applied in selected settings	[54,55]
ColoPrint	Tumor tissue	Gene Signature	Classifies stage II patients into low/high recurrence risk	Candidate tool for recurrence risk stratification	Under clinical validation	[56,57]

Abbreviations: miRNA, microRNA; lncRNA, long non-coding RNA; circRNA, circular RNA; mRNA, messenger RNA; PRC2, Polycomb repressive complex 2.

**Table 5:** Comparison of major proteomic platforms in CRC research. Different proteomic platforms provide complementary rather than interchangeable advantages in CRC research, and platform selection should be guided by the primary study objective, including discovery, cohort-scale validation, signaling analysis, or spatial characterization of the tumor microenvironment.

Platform	Main Strength	Main Limitation	Typical Application in CRC	Translational Suitability	Ref.
LC–MS/MS	Broad proteome coverage and strong unbiased discovery capability; applicable to tissue, plasma, and extracellular vesicle samples	Performance may be affected by sample complexity, instrument time, and batch effects	Global protein profiling, biomarker discovery, and pathway exploration	Moderate	[76]
TMT-based proteomics	High multiplexing capacity and efficient comparative analysis across multiple samples within the same batch	Ratio compression and co-isolation interference may affect quantitative accuracy; careful batch design and normalization are required	Comparative cohort studies, subtype analysis, and candidate biomarker prioritization	Moderate	[84]
DIA proteomics	Improved reproducibility, data completeness, and scalability for larger cohorts	Detection depth for some low-abundance proteins may depend on spectral libraries, acquisition settings, and computational workflow	Biomarker verification, large-cohort quantitative profiling, and translational validation studies	Moderate to high	[85]
Targeted phosphoproteomics	Functionally informative for pathway activation and drug-response signaling	Phosphopeptide enrichment and quantification are technically demanding; phosphorylation is highly context dependent	Signaling network analysis, therapeutic response assessment, and resistance mechanism studies	Moderate	[82]
IMC/MIBI (spatial proteomics)	Preserves tissue architecture and resolves tumor, stromal, and immune niches at high multiplexity	Higher cost, lower throughput, dependence on antibody panels, and complex image-analysis pipelines	Tumor microenvironment mapping, immune contexture analysis, and spatial biomarker discovery	Emerging	[83]

Abbreviations: LC–MS/MS, liquid chromatography–tandem mass spectrometry; TMT, tandem mass tag; DIA, data-independent acquisition; IMC, imaging mass cytometry; MIBI, multiplexed ion beam imaging.

**Table 7:** Metabolite biomarkers and clinical relevance in CRC. Representative metabolites associated with colorectal cancer, including tumor-related, lipid-associated, amino acid-related, and microbiota-derived metabolites, together with their sample type, metabolic pathway, functional roles in tumor progression, metabolic reprogramming, or immune modulation, and potential diagnostic, prognostic, or therapeutic relevance.

Metabolite	Sample Source	Pathway	Function in CRC	Clinical Relevance	Ref.
Lactate	Serum	Glycolysis	Supports rapid energy production and proliferation	Prognostic marker; therapy response indicator	[120]
Glutamine	Serum	Glutaminolysis	Provides carbon/nitrogen for biosynthesis	Predictive of metabolic dependencies	[121]
Succinate	Serum/plasma	TCA cycle	TCA-cycle-associated metabolite linked to immune regulation and tumor progression	Potential biomarker associated with CRC metabolic dysregulation and aggressive behavior	[122]
Ceramides	Plasma	Lipid metabolism	Promotes tumor signaling and membrane integrity	Potential lipidomic biomarker and therapeutic target candidate	[123]
Lysophosphatidylcholine	Plasma	Lipid metabolism	Correlates with tumor progression and inflammation	Diagnostic candidate for CRC detection	[124]
Kynurenine	Plasma/tumor tissue	Tryptophan catabolism	Mediates immune suppression and tumor immune evasion	Immunometabolic biomarker; associated with immune evasion and potential immunotherapy relevance	[125]
Butyrate	Stool/feces	SCFA/microbial	Exhibits anti-inflammatory and anti-tumor properties	Protective marker; reduced in CRC patients	[126]
Deoxycholic Acid (DCA)	Stool/serum	Bile acid metabolism	Induces DNA damage and promotes carcinogenesis	Risk-associated metabolite; potential marker of colorectal carcinogenesis	[127]
Sarcosine	Serum	One-carbon/methylation-related metabolism	Candidate metabolite reported in some diagnostic metabolomics studies	Exploratory biomarker	[128]
Tyrosine	Serum	Amino acid metabolism	Altered amino acid metabolite selected in CRC metabolic signatures	Part of serum metabolite panels for CRC detection	[121]

Abbreviations: TCA, tricarboxylic acid; SCFA, short-chain fatty acid; DCA, deoxycholic acid.

**Table 8:** Key CRC-associated microbes, sample type, mechanisms, and clinical relevance. Representative CRC-associated microbes, their major biospecimen source, mechanistic roles in tumorigenesis, study-specific associations, and potential diagnostic, prognostic, or therapeutic relevance.

Microbe	Sample Source	Main Mechanism	CRC Association	Potential Clinical Relevance	Ref.
*F. nucleatum* (CRC-associated clade)	Tumor tissue	Fad A–E-cadherin/β-catenin activation; biofilm formation; immune evasion via Fap2–TIGIT	Enriched in CRC tissue; a recent tumor-based study reported a CRC-associated clade in approximately half of tumor samples	Candidate diagnostic/prognostic biomarker and therapeutic target	[131]
ETBF (bft+)	Stool isolates	BFT; STAT3 and IL-17/Th17 inflammatory signaling	Detected in 38% of stool isolates from CRC patients versus 12% in controls in one clinical study	Risk-associated microbe and potential screening biomarker	[141]
pks+ *E. coli*	Tumor tissue/mucosal samples	Colibactin-mediated DNA damage and mutational signature formation	Linked to characteristic colibactin-associated mutational signatures in CRC and early tumorigenesis	Mechanistic biomarker and potential early-detection target	[142]
*Faecalibacterium prausnitzii*	Experimental CRC model/intestinal epithelial cells	Butyrate production; anti-inflammatory and epithelial-protective effects	Ameliorates colorectal tumorigenesis and suppresses CRC cell proliferation in preclinical studies	Protective/probiotic candidate	[143,144]

Abbreviations: ETBF, enterotoxigenic *Bacteroides fragilis*; BFT, *B. fragilis* toxin; STAT3, signal transducer and activator of transcription 3; IL-17, interleukin 17; TIGIT, T cell immunoreceptor with Ig and ITIM domains.

**Table 9:** Representative integrative multi-omics studies in colorectal cancer. This table summarizes selected multi-omics studies in CRC, highlighting the integrated omics layers and their main biological or clinical applications. Collectively, these studies show that multi-omics integration can improve molecular subtyping, treatment-response prediction, and the understanding of tumor-host-microbiota interactions. They also support the development of machine learning-based precision oncology approaches.

Integrated Omics Layers	Main Application	Key Finding	Representative Study
Genomics + Transcriptomics	Consensus molecular subtyping	Established biologically distinct CRC subtypes with prognostic and therapeutic relevance	[69]
Genomics + Transcriptomics + Proteomics + Phosphoproteomics	Tumor classification and therapy prediction	Improved functional stratification by identifying protein activity and signaling states not captured by mRNA expression alone	[160]
Metagenomics + Transcriptomics + Metabolomics	Analysis of tumor-microbiota-host interactions	Revealed how microbiome-associated metabolic and host transcriptional changes shape the tumor microenvironment	[161]
Proteogenomics + Immune Profiling	Immune phenotype classification in CRC	Identified immune-related CRC subsets with distinct microenvironmental features and potential differences in immunotherapy response	[162]
Multi-omics + Machine learning	Prognosis prediction and CRC subtype modeling	Enhanced risk stratification and predictive modeling by integrating heterogeneous molecular features	[163]

Abbreviations: CMS, Consensus Molecular Subtypes; mRNA, messenger RNA.

## Data Availability

No new data were created or analyzed in this study.

## Abbreviations

The following abbreviations are used in this manuscript:

AI	artificial intelligence
AUC	area under the curve
BFT	Bacteroides fragilis toxin
BRAF	B-Raf proto-oncogene
CA19-9	carbohydrate antigen 19-9
CEA	carcinoembryonic antigen
CE-MS	capillary electrophoresis-mass spectrometry
cfRNA	cell-free RNA
CIMP	CpG island methylator phenotype
CIN	chromosomal instability
circRNA	circular RNA
CMS	Consensus Molecular Subtypes
CPTAC	Clinical Proteomic Tumor Analysis Consortium
CRC	colorectal cancer
CRISPR-Cas9	clustered regularly interspaced short palindromic repeats-associated protein 9
ctDNA	circulating tumor DNA
DEGs	differentially expressed genes
DIA	data-independent acquisition
DIABLO	Data Integration Analysis for Biomarker discovery using Latent cOmponents
dMMR	deficient mismatch repair
EGF	epidermal growth factor
EGFR	epidermal growth factor receptor
EMT	epithelial–mesenchymal transition
EOCRC	early-onset colorectal cancer
ESMO	European Society for Medical Oncology
ETBF	enterotoxigenic Bacteroides fragilis
FDA	U.S. Food and Drug Administration
FMT	fecal microbiota transplantation
GC-MS	gas chromatography-mass spectrometry
GNNs	graph neural networks
H2S	hydrogen sulfide
HDAC	histone deacetylase
HER2	human epidermal growth factor receptor 2
IL-6	interleukin 6
IL-17	interleukin 17
IMC	imaging mass cytometry
KRAS	Kirsten rat sarcoma viral oncogene homolog
LAG-3	lymphocyte-activation gene 3
LC-MS	liquid chromatography-mass spectrometry
LC-MS/MS	liquid chromatography-tandem mass spectrometry
LDHA	lactate dehydrogenase A
LIME	Local Interpretable Model-agnostic Explanations
lncRNA	long non-coding RNA
MAPK	mitogen-activated protein kinase
MEK	MAPK/ERK kinase
MET	MET proto-oncogene, receptor tyrosine kinase
MIBI	multiplexed ion beam imaging
miRNA	microRNA
MLH1	MutL homolog 1
MMR	mismatch repair
MOFA	Multi-Omics Factor Analysis
MSI	microsatellite instability
MSI-H	microsatellite instability-high
MYC	MYC proto-oncogene
NCCN	National Comprehensive Cancer Network
NF-κB	nuclear factor kappa B
NF1	neurofibromin 1
NMR	nuclear magnetic resonance
NTRK	neurotrophic tyrosine receptor kinase
OS	overall survival
PD-1	programmed cell death protein 1
PD-L1	programmed death-ligand 1
PD-L2	programmed death-ligand 2
PI3K	phosphatidylinositol 3-kinase
PIK3CA	phosphatidylinositol-4,5-bisphosphate 3-kinase catalytic subunit alpha
PRC2	Polycomb repressive complex 2
PTEN	phosphatase and tensin homolog
PTMs	post-translational modifications
PDOs	patient-derived organoids
RFS	relapse-free survival
RNA-seq	RNA sequencing
SCFA	short-chain fatty acid
SCNAs	somatic copy number alterations
scRNA-seq	single-cell RNA sequencing
SHAP	Shapley Additive exPlanations
SMAD4	SMAD family member 4
SNF	similarity network fusion
STAT3	signal transducer and activator of transcription 3
TCA	tricarboxylic acid
TCGA	The Cancer Genome Atlas
TGF-β	transforming growth factor beta
TGFBR2	transforming growth factor beta receptor 2
TH1	T helper 1
TIGIT	T cell immunoreceptor with Ig and ITIM domains
TMT	Tandem Mass Tag
TP53	tumor protein p53
VEGF-A	vascular endothelial growth factor A
WNT	Wingless/Integrated signaling pathway
XAI	explainable artificial intelligence
